# Moment Redistribution in Continuous Externally CFRP Prestressed Beams with Steel and FRP Rebars

**DOI:** 10.3390/polym13081181

**Published:** 2021-04-07

**Authors:** Tiejiong Lou, Zhangxiang Li, Miao Pang

**Affiliations:** 1 Hubei Key Laboratory of Roadway Bridge & Structure Engineering, Wuhan University of Technology, Wuhan 430070, China; tjlou@whut.edu.cn (T.L.); whutlizx@163.com (Z.L.); 2 Department of Civil Engineering, Zhejiang University, Hangzhou 310058, China

**Keywords:** carbon fiber, glass fiber, external tendon, moment redistribution, finite element

## Abstract

This paper assesses the impact of adopting carbon- or glass-fiber-reinforced polymer (CFRP or GFRP) instead of steel rebars on the redistribution of moments in prestressed concrete beams (PCBs) with external CFRP tendons. A numerical program is introduced, and numerical simulations are performed on two-span continuous beams with steel, CFRP or GFRP rebars of various areas, i.e., Ar2 = 360–3560 mm^2^, and Ar1/Ar2 = 1.5, where Ar1 and Ar2 are areas of tensile rebars over the positive and negative moment zones, respectively. The results show the moment redistribution is contributed by concrete cracking only for the beams with fiber-reinforced polymer (FRP) rebars, and by concrete cracking and steel yielding for the beams with steel rebars. As a result, the use of FRP rebars leads to a substantially lower moment redistribution than in steel rebars. It is also demonstrated that Eurocode 2, CSA A23.3-04 and ACI 318-19 fail to reflect the rebar influence on moment redistribution in PCBs with external tendons. A simplified equation for the quantification of moment redistribution in externally PCBs with steel and FRP rebars is recommended, which yields accurate and conservative predictions.

## 1. Introduction

The use of external prestressing offers many advantages such as the ease of tendon inspection and replacement, flexible choice of cross-section of structures, reduction in dead load by permitting thinner web, and low friction loss [1]. As such, external tendons are extensively employed for the strengthening of engineering structures, especially continuous bridges. The non-corrosive fiber-reinforced polymer (FRP) is a promising alternative to prestressing steel tendons [2,3]. Various FRPs are available in civil engineering applications [4,5,6,7,8], e.g., aramid, carbon and glass FRPs (AFRP, CFRP and GFRP). Among those, CFRP shows the best resistance to creep rupture and is particularly suitable for prestressing applications. The use of external CFRP tendons to replace external steel tendons has been proven to be feasible without compromising the workability of the structure [9,10,11].

Moment redistribution in continuous prestressed concrete beams (PCBs) needs to be carefully considered for an economical and safe structural design. A small number of works have been performed to evaluate the redistribution behavior of PCBs with external tendons. Aravinthan et al. [12] tested six two-span PCBs with external steel tendons under symmetrical or unsymmetrical loading. They concluded that symmetrical loading led to positive redistribution of moments in the support section, and negative one in the midspan section, and that moment redistribution under unsymmetrical loading was insignificant [12]. We should note that this conclusion has resulted from the particular reinforcement arrangement of the specimens. Moment redistribution in a critical section might be positive or negative at symmetrical loading, and might be important or unimportant at unsymmetrical loading, depending on the arrangement of bonded reinforcements [13]. The experimental results by Chan and Au [14] indicated that neither the neutral axis depth, nor net strain in the extreme tensile reinforcement correlated well with the amount of moment redistribution in externally PCBs, confirming that moment redistribution is member-dependent. Results obtained from numerical simulations led to similar observations [15,16]. A parametric study was conducted to investigate various parameters influencing moment redistribution at the ultimate limit state in PCBs with external CFRP tendons [15]. The strengthening of reinforced concrete beams (RCBs) by external prestressing resulted in a significant decrease in moment redistribution [15]. Several code equations that adopted the neutral axis depth for quantifying moment redistribution were modified by introducing a key parameter representing the stiffness difference [16]. Lou et al. [17] found that moment redistribution at the center support was substantially reduced by an upward linearly transformed movement of external cables, while the influence of linear transformation on moment redistribution over the midspan was marginal.

All of the aforementioned works were focused on moment redistribution in externally PCBs with steel rebars. In externally post-tensioned members, the provision of a certain amount of bonded rebars is required to ensure favorable flexural performance and crack pattern [18]. Bonded rebars play a vital role in the structural behavior and moment redistribution in these members. Conventional steel rebars are subject to corrosive damage, which can be overcome by replacement with FRP rebars [19], e.g., CFRP and GFRP. Many works have been performed to investigate the feasibility of using FRP rebars in concrete elements, especially when exposed to a harsh environment [20,21,22,23,24]. A recent study showed that simply supported externally PCBs with FRP rebars exhibited significantly different behavior from that of those with steel rebars, including crack pattern, load-deformation characteristics, and stress in external tendons [25]. The brittleness of FRP rebars would raise concerns around their ability to redistribute moments in continuous beams. While extensive works on continuous FRP RCBs have been performed [26,27,28,29,30,31,32], the effect of adopting FRP rebars instead of those made of steel on moment redistribution in continuous externally PCBs has not yet been addressed.

This study presents a comparative study on the use of FRP and steel rebars in continuous PCBs with external CFRP tendons, focusing on the behavior related to the redistribution of moments, and a numerical program is introduced. Numerical simulations are then conducted on two-span continuous beams to investigate the effect of adopting FRP rebars instead of steel ones on the redistribution behavior. Moreover, several codes of practice are assessed, and a reasonable recommendation for quantifying moment redistribution in externally PCBs with steel and FRP rebars is made.

## 2. Numerical Program

A numerical program considering geometrical and material nonlinearity has been developed [33]. The geometrical nonlinearity was introduced by continuously updating the effective depth of external tendons and also by coupling flexural and axial fields. The nonlinear constitutive laws of materials were introduced in the numerical procedure by utilizing the layered method. The finite elements were formulated by applying the Euler–Bernoulli theory. The contribution of external prestressing was made with equivalent loads. Detailed numerical treatment on beam elements and prestressing effect can be referred to [33].

**Figure 1:**
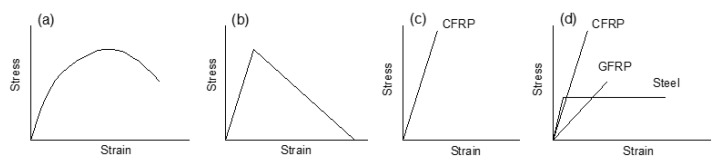
Schematic diagrams of material laws. (**a**) concrete in compression; (**b**) concrete in tension; (**c**) tendons; (**d**) rebars.

Figure 1 illustrates the laws of constituent materials adopted in this study, namely, the stress–strain law suggested by Eurocode 2 [34] for concrete in compression, an elastic and tension–stiffening law for concrete in tension, a linear–elastic law for FRP tendons and rebars [1,19], and an elastic–perfectly plastic law for steel rebars [18].

An incremental method, together with the Newton–Raphson iterative algorithm, was used to solve the nonlinear equilibrium equations. The iterations at every increment involved several steps, i.e., forming the tangent stiffness matrix, solving the equilibrium equations, determining the state of elements and checking the convergence. The numerical program is able to simulate the complete response of continuous PCBs with external tendons throughout the loading history, from prestressing until the ultimate limit state. The validation of the numerical program has been reported in [13,17,35], where the numerical predictions were compared against the experimental data of a number of continuous PCBs with external tendons and the comparisons showed favorable agreement.

## 3. Numerical Investigation

**Figure 2:**
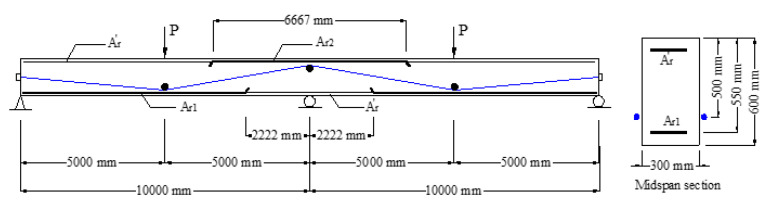
Continuous beam for numerical investigation.

A two-span continuous PCB with external tendons, as shown in Figure 2, is used. Each span has a length of 10.0 m and is subjected to a concentrated load at the midspan. The rectangular section is 300 mm in width and 600 mm in depth. CFRP composites are used as external tendons, with an area of 1000 mm^2^, elastic modulus of 147 GPa, and a rupture strength of 1840 MPa. The initial prestress is 1104 MPa. The tendon eccentricities at the end support, midspan and center support are 0, 140 and 140 mm, respectively. The areas of tensile rebars at the positive and negative moment zones, *A_r_*_1_ and *A_r_*_2_, are variables, and the *A_r_*_1_/*A_r_*_2_ ratio is fixed at 1.5. The value of *A_r_*_2_ varies from 360 to 3560 mm^2^, i.e., the ratio of tensile rebars at the center support, *ρ_r_*_2_ = *A_r_*_2_/(*bd_r_*_2_), ranges from 0.22% to 2.16%, where *b* is section width, and *d_r_*_2_ is the depth of tensile rebars at the center support. The area of compressive rebars, $A_{r}^{\prime }$, is 360 mm^2^. The rebars are made of steel (yield strength of 450 MPa and elastic modulus of 200 GPa), CFRP (rupture strength of 1840 MPa and elastic modulus of 147 GPa), or GFRP (rupture strength of 750 MPa and elastic modulus of 40 GPa). The concrete cylinder compressive strength, tensile strength and elastic modulus are 60 MPa, 4.4 MPa and 39 GPa, respectively.

### 3.1. Support Reaction and Bending Moment

**Figure 3:**
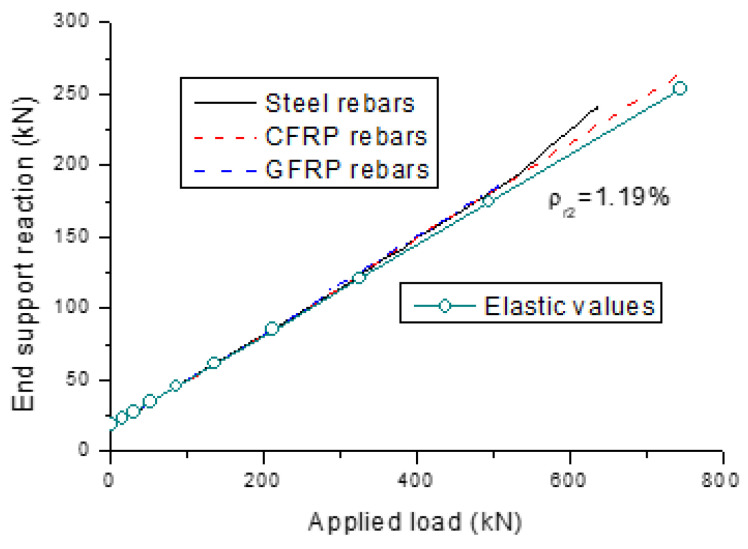
Development of end support reactions for the beams with different types of rebars.

**Figure 4:**
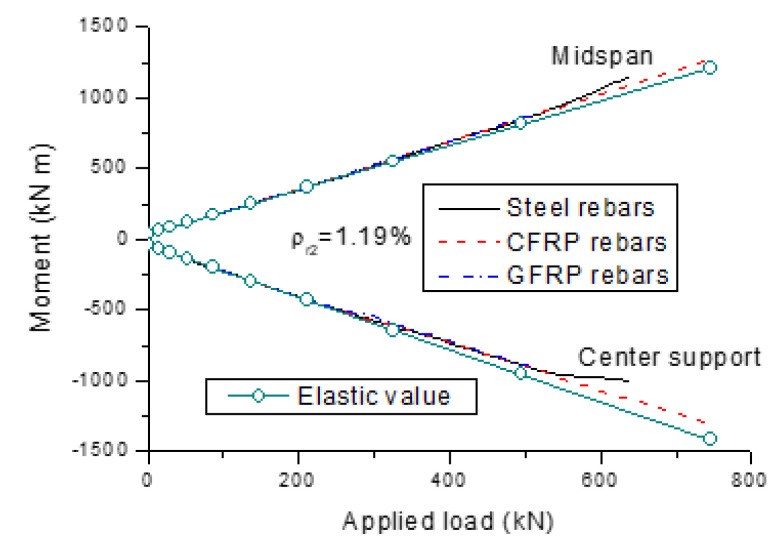
Development of bending moments for the beams with different types of rebars.

Figure 3 and Figure 4 show the development of end support reactions and bending moments for the beams with different types of rebars, respectively. The results are generated for a *ρ_r_*_2_ of 1.19%. The elastic values obtained from the analysis, assuming linearly elastic properties of materials, are also plotted. The reactions or moments illustrated in the graphs consist of three components, which were induced by dead load, applied load and external prestressing, respectively. We see that the external cables are slightly below their concordant line, leading to a small upward secondary reaction at the end support and, correspondingly, small positive secondary moments along the span.

We see that the actual reaction or moment does not deviate from the elastic value until the cracking load is reached. Further to this, the load versus reaction or moment relationship for the beams with FRP rebars exhibits approximately a linear manner up to failure. Prior to steel yielding, the load versus reaction or moment behavior for the beam with steel rebars is very similar to that with FRP rebars. When the steel rebars at the center support begin to yield, moments are redistributed from the center support to the midspan. As a consequence, there appears to be a faster increase in the reaction at the end support, as shown in Figure 3. Correspondingly, the positive moment at the midspan grows quicker and the negative moment at the center support grows slower, as shown in Figure 4.

**Figure 5:**
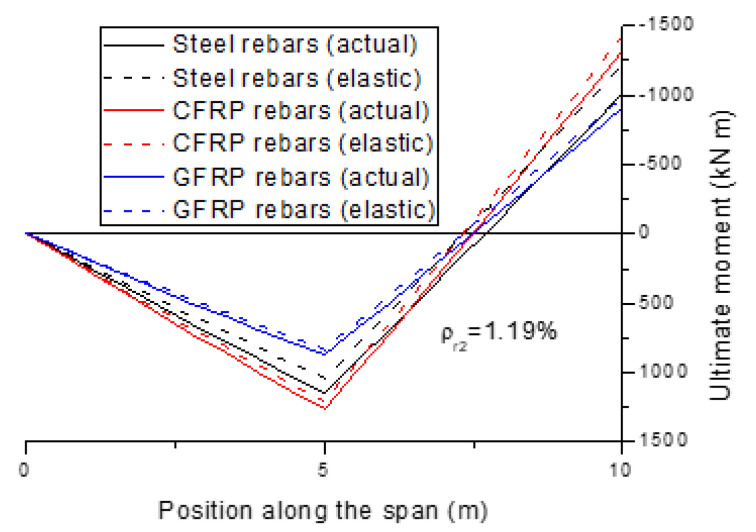
Moment distribution for the beams with different types of rebars.

Figure 5 shows the moment distribution at the ultimate limit state for the beams with different types of rebars (*ρ_r_*_2_ = 1.19%). We see that at the center support, the actual moment is smaller than the elastic moment, leading to a positive redistribution of moments, while the phenomenon is the opposite at the midspan. Moreover, the difference between the actual and elastic moments in the beam with FRP rebars is slight, indicating an insignificant redistribution of moments. Conversely, the difference in the beam with steel rebars is substantial, especially at the center support, indicating a notable redistribution of moments.

### 3.2. Reaction Ratio and Moment Ratio

Denote by *R*_1_ and *R*_2_ the load induced actual reactions at the end and center supports, respectively; by *R_e_*_1_ and *R_e_*_2_, the load induced elastic reactions at the end and center supports, respectively; by *M*_1_ and *M*_2_, the load induced actual moments at the midspan and center support, respectively; by *M_e_*_1_ and *M_e_*_2_, the load induced elastic moments at the midspan and center support, respectively. During the loading process, the *R_e_*_2_/*R_e_*_1_ or *M_e_*_2_/*M_e_*_1_ ratio for a continuous beam remains constant according to elastic theory, while the *R*_2_/*R*_1_ or *M*_2_/*M*_1_ ratio would vary when redistribution of moments occurs.

**Figure 6:**
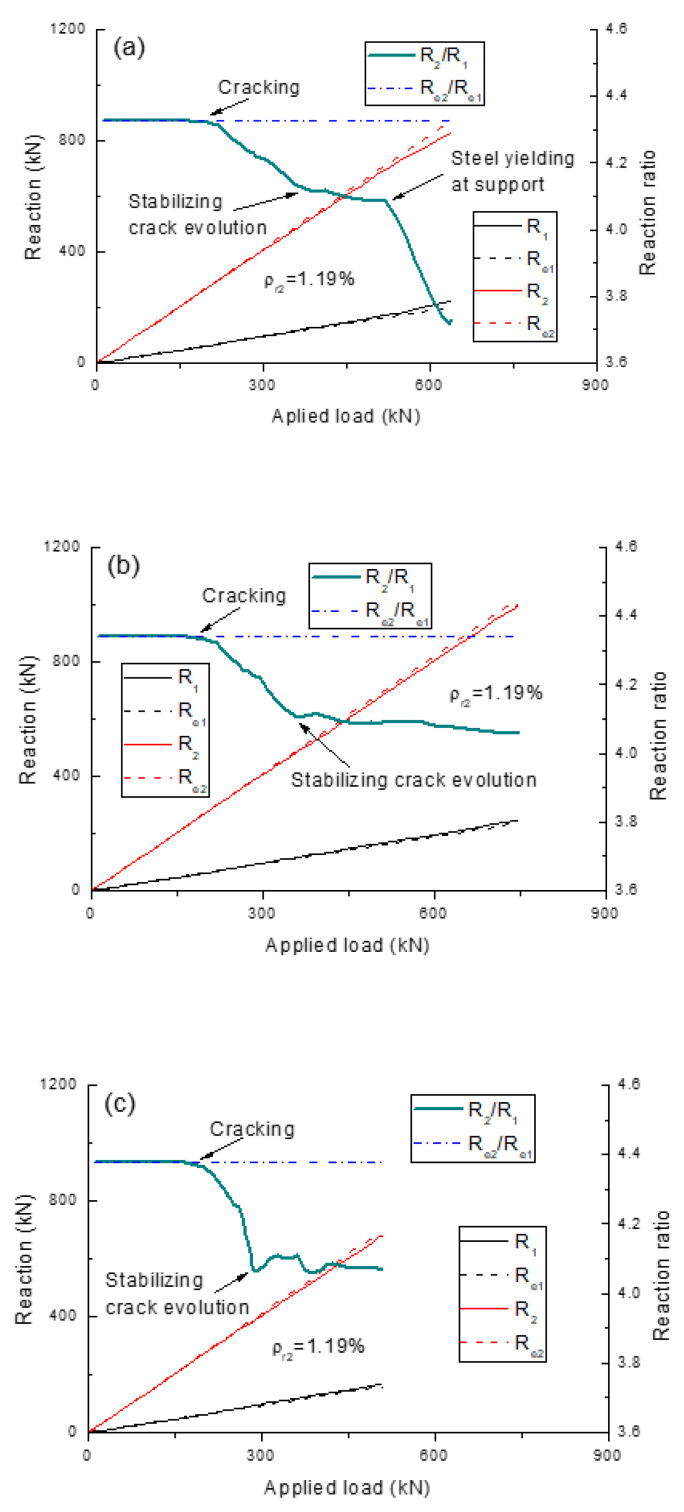
Development of reaction ratios for the beams with different types of rebars. (**a**) steel rebars; (**b**) carbon-fiber-reinforced polymer (CFRP) rebars; (**c**) glass-fiber-reinforced polymer (GFRP) rebars.

**Figure 7:**
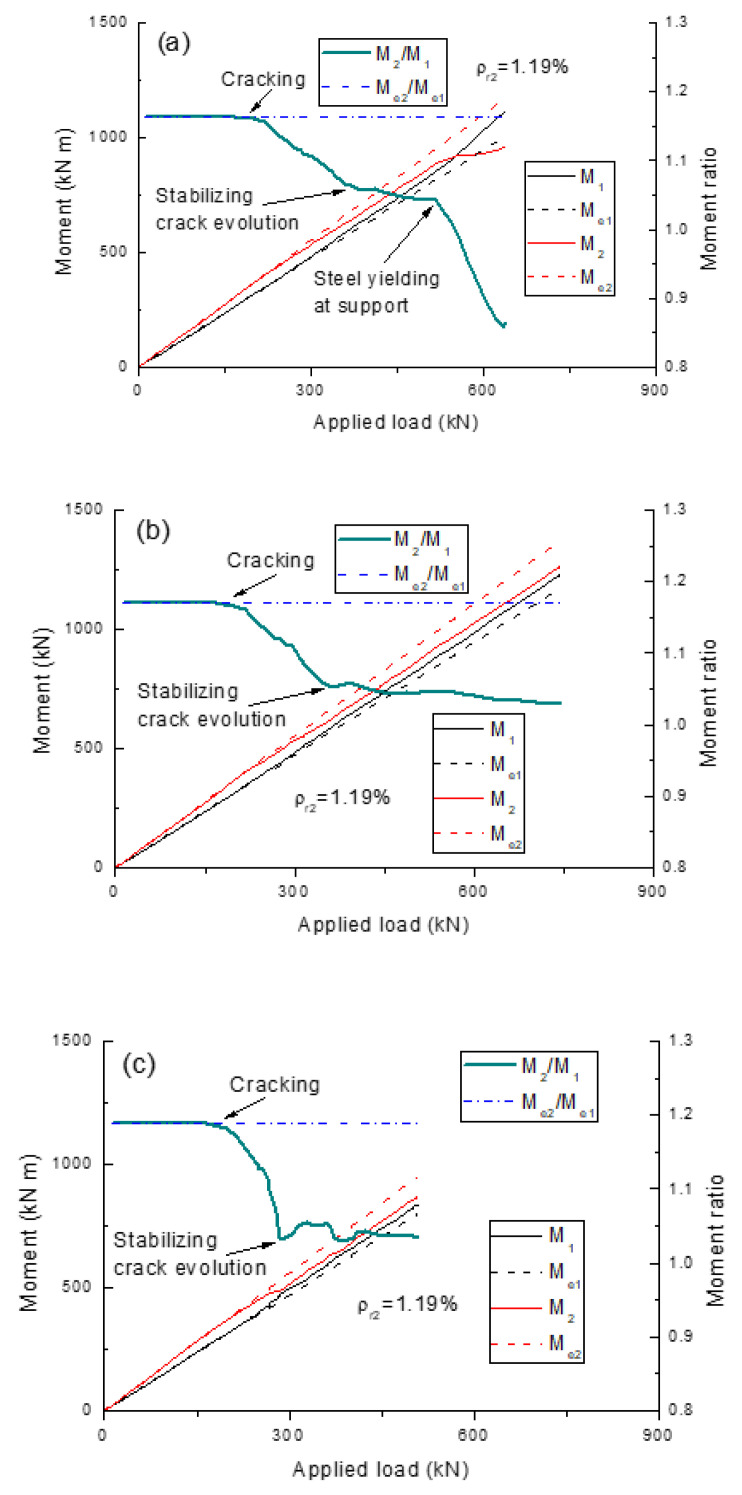
Development of moment ratios for the beams with different types of rebars. (**a**) steel rebars; (**b**) CFRP rebars; (**c**) GFRP rebars.

Figure 6 shows the evolution of load-induced reactions and reaction ratios for the beams with different types of rebars (*ρ_r_*_2_ = 1.19%), while the development of load-induced moments and moment ratios are presented in Figure 7. The results confirm that the elastic reaction ratio, or moment ratio, remains unchanged despite the load level. The *R_e_*_2_/*R_e_*_1_ ratios for the beams with steel, CFRP and GFRP rebars are 4.33, 4.34 and 4.38, respectively, while those of *M_e_*_2_/*M_e_*_1_ are 1.16, 1.17 and 1.19, respectively. This slight difference is attributed to the different contributions of the rebars to the transformed section. On first cracking, moments are redistributed from the center support towards the midspan, leading to slower development of *R*_2_ or *M*_2_, and faster development of *R*_1_ or *M*_1_ compared to their elastic values. Consequently, the *R*_2_/*R*_1_ or *M*_2_/*M*_1_ ratio begins to decrease. When the crack development stabilizes, the actual reaction or moment ratio for the beams with FRP rebars tends to stabilize until their ultimate failure. For the beams with steel rebars, the yielding of steel rebars leads to further moment redistribution from the center support to the midspan, causing a further decrease in the *R*_2_/*R*_1_ or *M*_2_/*M*_1_ ratio.

### 3.3. Degree of Moment Redistribution

**Figure 8:**
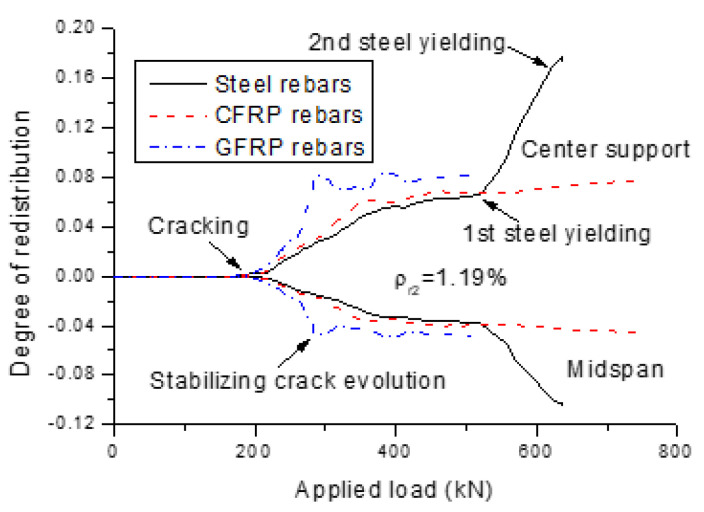
Load versus moment redistribution curves for the beams with different types of rebars.

Figure 8 shows the development of moment redistribution, with increasing load for the beams with different types of rebars (*ρ_r_*_2_ = 1.19%). The degree of redistribution is defined as $\beta =1-M/M_{e}$, where *M* is the actual moment and *M_e_* is the elastic moment. Moment redistribution does not happen until the occurrence of first cracking. After cracking, the degree of redistribution increases quickly. When the redistribution for the beams with FRP rebars reaches a plateau, there is a tendency to stabilize up to failure. The beam with steel rebars exhibits similar redistribution behavior to that of the beams with FRP rebars up to first steel yielding, and thereafter resumes a quick redistribution development.

**Figure 9:**
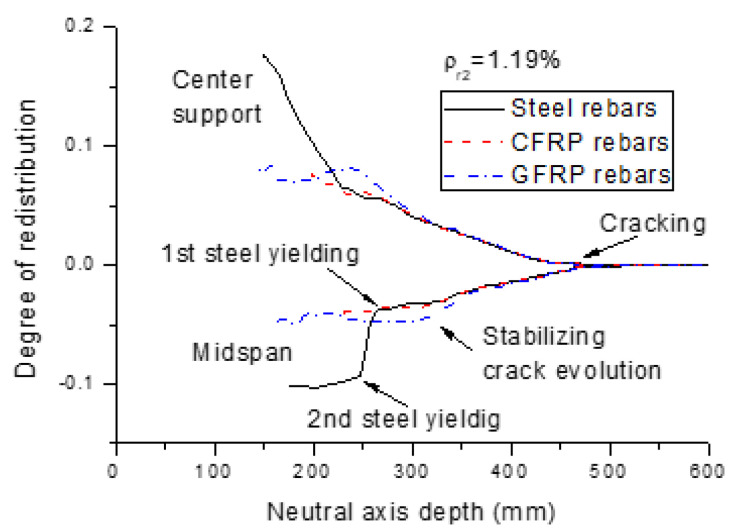
Neutral axis depth versus moment redistribution curves for the beams with different types of rebars.

**Figure 10:**
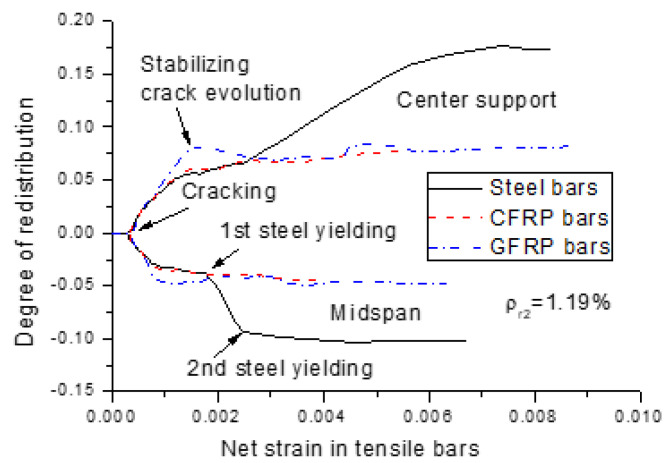
Rebar strain versus moment redistribution curves for the beams with different types of rebars.

Moment redistribution relies strongly on the ductility described by either the neutral axis depth, or net strain in tensile rebars. Figure 9 and Figure 10 show the moment redistribution versus neutral axis depth, and net strain in tensile rebars curves for the beams with different types of rebars (*ρ_r_*_2_ = 1.19%), respectively. The curves comprise three distinct stages for the beams with FRP rebars, while there are two additional stages for the beams with steel rebars. The first stage corresponds to the elastic stage with zero moment redistribution. In this stage, the neutral axis shifts rapidly from infinity towards the extreme compressive fiber of the section, while the rebar strain is marginal. In the second stage, moment redistribution develops linearly with decreasing neutral axis depth or increasing rebar strain until the crack evolution stabilizes. In these two stages, the beams with FRP rebars exhibit approximately the same behavior to that of the beams with steel rebars. The third stage is characterized by stabilizing redistribution. For the beams with FRP rebars, this stage continues until failure, accompanied by a substantial variation in neutral axis depth and rebar strain. For the beams with steel rebars, the fourth stage, triggered by the yielding of steel bars at the center support, is characterized by a quick development of moment redistribution with decreasing neutral axis depth or increasing rebar strain. The fifth stage, triggered by the yielding of steel bars at the midspan, is featured by stabilizing the redistribution of moments with varying neutral axis depth, or rebar strain up to the ultimate limit state.

**Figure 11:**
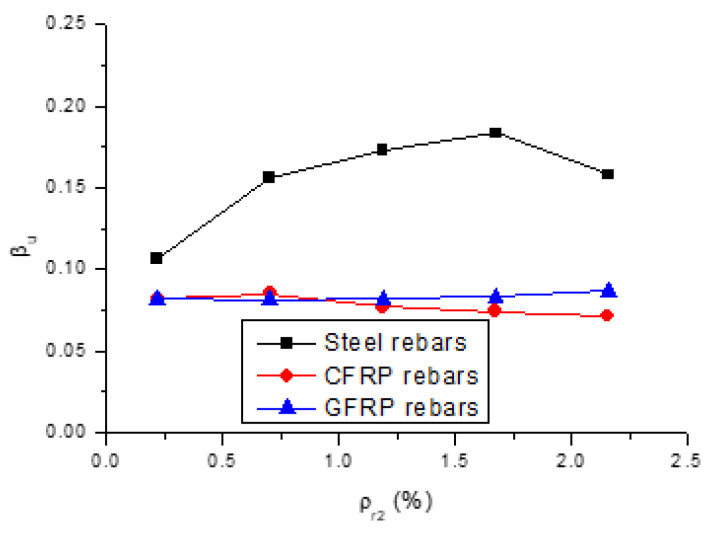
Variation in βu with varying ρr2 for the beams with different types of rebars.

The change in the value of *β_u_* (degree of redistribution at the ultimate limit state) over the center support with the *ρ_r_*_2_ level is displayed in Figure 11. We see that the *β_u_* value for the beams with GFRP rebars stabilizes around 8%, with varying *ρ_r_*_2_. The *β_u_* value for the beams with CFRP rebars slightly increases with increasing *ρ_r_*_2_ up to 0.7%, and thereafter turns to decrease slightly. In general, moment redistribution in the beams with CFRP rebars is very close to that in the beams with GFRP rebars. For the beams with steel rebars, the *β_u_* value increases with increasing *ρ_r_*_2_ up to 1.67%. Thereafter, a higher *ρ_r_*_2_ level results in a lower value of *β_u_*. This phenomenon can be explained by the fact that the amount of rebars influences both the ductility, and relative stiffness, between the critical positive and negative moment zones. As *ρ_r_*_2_ increases, the ductility of the center support section decreases, leading to a decrease in moment redistribution. Meanwhile, a higher *ρ_r_*_2_ gives rise to a larger stiffness difference between the center support and midspan, leading to a higher degree of redistribution. Therefore, the variation in the *β_u_* value with varying *ρ_r_*_2_ depends on the combined effects of ductility and relative stiffness.

It is also seen in Figure 11 that moment redistribution in the beams with steel rebars is substantially higher than that seen in the beams with FRP rebars, which is attributed to the notable contribution of steel yielding. The difference between the *β_u_* values for the beams with steel and FRP rebars tends to enlarge as *ρ_r_*_2_ increases up to 1.67%, and narrows thereafter. For *ρ_r_*_2_ = 0.22%, 1.67% and 2.16%, the redistribution values mobilized by steel rebars are 1.3, 2.5 and 2.2 times, respectively, those by CFRP rebars and 1.3, 2.2 and 1.8 times, respectively, those by GFRP rebars.

## 4. Theoretical Consideration

### 4.1. Current Design Codes for Calculating Moment Redistribution

While several codes or guides, e.g., ACI 440.1R-06 [19] and ACI 440.4R-04 [1], which deal with FRP bars/tendons are available, they do not provide specific rules for moment redistribution in continuous beams reinforced with FRP bars, or those prestressed with FRP tendons. It has been shown that PCBs with external CFRP and steel tendons exhibited similar redistribution behavior [17]. Conversely, the redistribution behavior of the beams with FRP rebars differs significantly from that of the beams with steel rebars, as discussed in previous sections. Therefore, it is worth investigating whether the current codes for RCBs or PCBs are applicable to cases whereby FRP bars/tendons are used. Three codes of practice are considered, namely, Eurocode 2 [34], CSA A23.3-04 [36] and ACI 318-19 [18]. These codes adopted either the neutral axis depth [34,36] or net strain in extreme tensile reinforcement [18] as a key parameter for calculating the allowable moment redistribution in RCBs or PCBs.

Eurocode 2 [34] recommended the following equation for calculating the permissible moment redistribution

(1) $$\beta_{u}=C-1.25\left(0.6+0.0014/\varepsilon_{u}\right)c_{u}/d$$

where *d* is the section effective depth; *ε_u_* is the concrete ultimate compressive strain; *C* is a coefficient depending on the concrete grade, i.e., *C* = 0.56 for normal-strength concrete and 0.46 for high-strength concrete. The redistribution limit specified by Eurocode 2 is 30% for sufficiently ductile reinforcement, and 20% for insufficiently ductile reinforcement.

CSA A23.3-04 [36] suggested that the value of elastic moments over the supports could be adjusted by

(2) $$\beta_{u}=0.3-0.5c_{u}/d$$

where the redistribution limit specified by CSA A23.3-04 is 20%.

ACI 318-19 [18] used the following expression for calculating the permissible redistribution

(3) $$\beta_{u}=\left(1000\varepsilon_{t}\right)\%$$

where *ε_t_* is the net tensile strain in the extreme layer of longitudinal tension reinforcement at the ultimate limit state, excluding pre-strain due to effective prestressing. The value of *ε_t_* should not be lower than 0.0075. The redistribution limit specified by ACI 318-19 is 20%.

### 4.2. Evaluation of Design Codes

**Figure 12:**
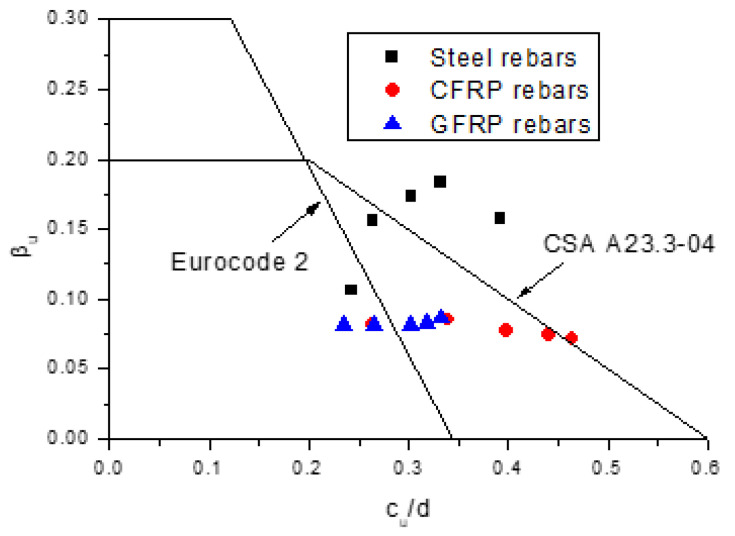
Variation in *β_u_* with varying *c_u_*/*d* for the beams with different types of rebars along with the code curves (Eurocode 2 and CSA A23.3-04).

Figure 12 shows the numerically obtained data regarding the *c_u_*/*d*-*β_u_* relationship along with the code curves (Eurocode 2 and CSA A23.3-04). According to the numerical analysis, the *β_u_* value for the beams with steel rebars is increased substantially by 72.45% when varying *c_u_*/*d* from 0.24 to 0.33. In contrast, the beams with FRP rebars exhibit a stabilizing redistribution at the ultimate limit state regardless of the value of *c_u_*/*d*. However, such observations from numerical simulations are either opposite to, or inconsistent with the design codes, as the latter exhibits a trend of decrease in *β_u_* with increasing *c_u_*/*d*. Therefore, both codes cannot reflect the trend regarding the variation in *β_u_* with varying *c_u_*/*d*. It is also observed in the figure that most of the data lie beyond the Eurocode 2 curve, while below the CSA A23.3-04 curve, indicating conservative predictions by Eurocode 2 but non-conservative predictions by CSA A23.3-04.

**Figure 13:**
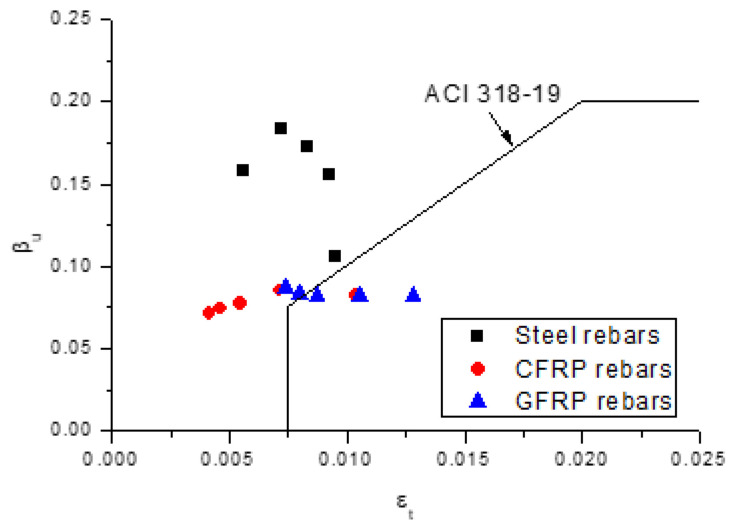
Variation in *β_u_* with varying *ε_t_* for the beams with different types of rebars along with the ACI 318-19 curve.

Figure 13 illustrates the numerically obtained data regarding the *ε_t_*-*β_u_* relationship along with the ACI 318-19 curve. We see that, as far as the variation in *β_u_* with varying *ε_t_* is concerned, ACI 318-19 fails to reflect the actual tendency for the beams with steel or FRP rebars. In addition, all of the data for the beams with steel rebars are above the code curve, demonstrating safe predictions of ACI 318-19. For the beams with FRP rebars, some data are below the code curve, implying unsafe predictions of ACI 318-19.

**Figure 14:**
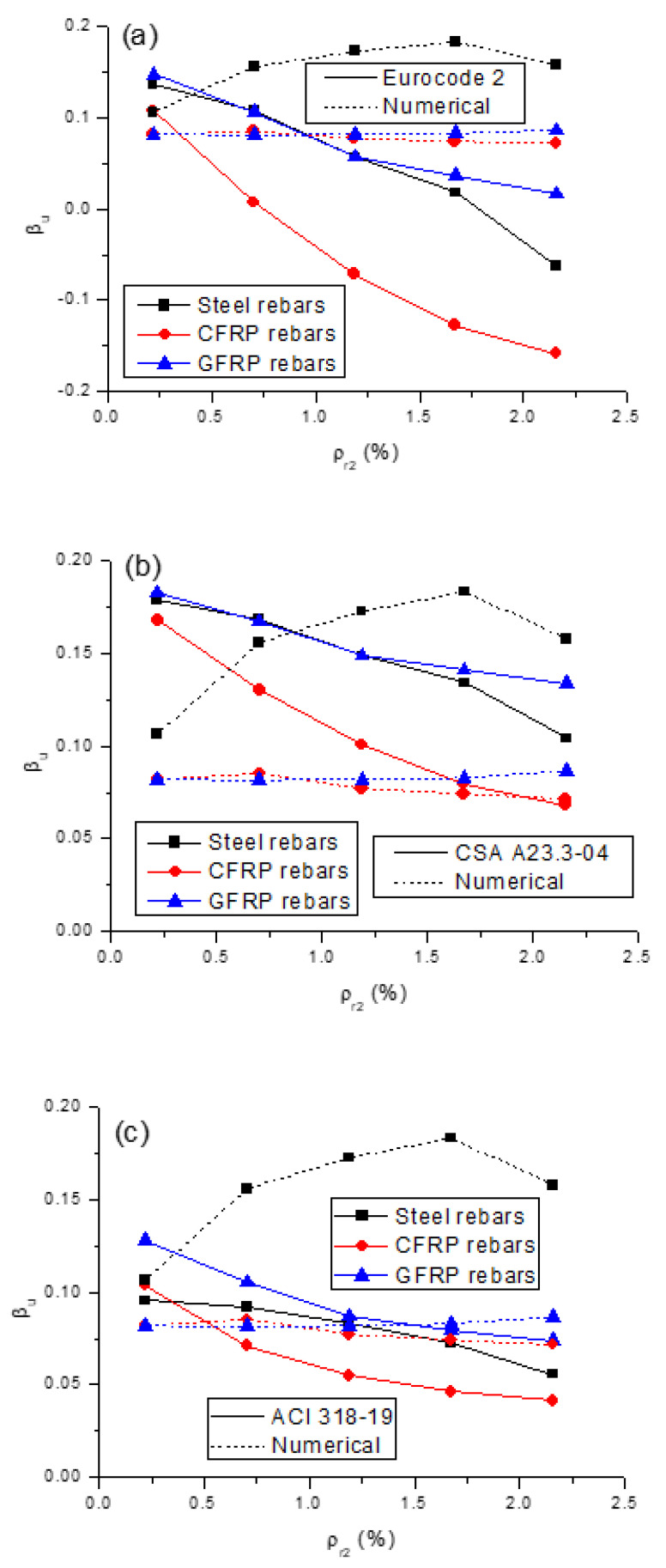
Comparison of numerically predicted *β_u_* values and code predictions. (**a**) Eurocode 2; (**b**) CSA A23.3-04; (**c**) ACI 318-19.

A comparison of the *β_u_*-*ρ_r_*_2_ relationship predicted by the design codes, and numerical analysis for the beams with different types of rebars, is given in Figure 14. According to the design codes, the *β_u_* value for the beams with steel or FRP rebars consistently decreases as *ρ_r_*_2_ increases. However, this is not concordant with the numerical prediction regarding the *β_u_*-*ρ_r_*_2_ relationship, as seen in Figure 14. This can be attributed to the fact that the design codes account for the section ductility only, neglecting the influence of relative stiffness, while both ductility and relative stiffness are affected by the amount of rebars. As a consequence, the influence of rebar amount on moment redistribution could not be reasonably reflected in the design codes. In addition, according to the code prediction, the redistribution for the beams with CFRP rebars is substantially lower than that for the beams with GFRP rebars. The redistribution values for the beams with GFRP rebars are almost identical to, or higher, than that for the beams with steel rebars. The aforementioned observations are also inconsistent with actual (i.e., numerically predicted) influence of the type of rebars on moment redistribution. Therefore, the design codes could not reflect the influence of rebars (both the amount and type) on the moment redistribution in externally PCBs.

We can also observe from Figure 14 that Eurocode 2 is generally conservative for the beams with CFRP or steel rebars; however, it might be non-conservative for the beams with GFRP rebars at low *ρ_r_*_2_ levels (*ρ_r_*_2_ < 0.95%). CSA A23.3-04 is unsafe when predicting the moment redistribution in the beams with FRP rebars. When steel rebars are used, CSA A23.3-04 is not safe at *ρ_r_*_2_ < 0.86%. ACI 318-19 is conservative for the beams with steel rebars, whereas it appears to be unsafe for the beams with CFRP rebars at *ρ_r_*_2_ < 0.52% and for the beams with GFRP rebars at *ρ_r_*_2_ < 1.49%.

### 4.3. Recommended Equation

As illustrated in Figure 11, the *β_u_* value for the beams with FRP rebars is around 8% regardless of the type and amount of FRP rebars. Therefore, a redistribution value of 8% may be used in the design of continuous externally PCBs with FRP rebars. For the beams with steel rebars, a modified CSA A23.3-04 equation with a new coefficient *λ_csa_* reflecting the relative stiffness of critical sections [16] may be adopted. Hence, the following equation is recommended to predict the moment redistribution at the ultimate limit state in externally PCBs with steel and FRP rebars

(4) $$\beta_{u}=\{\begin{array}{c} \lambda_{csa}\left(0.3-0.5c_{u}/d\right)\;\mathrm{for}\;\mathrm{steel}\;\mathrm{rebars}\\ 8\%\;\mathrm{for}\;\mathrm{FRP}\;\mathrm{rebars} \end{array}$$

where

(5) $$\lambda_{csa}=0.43+2.71ln(\omega_{1}/\omega_{2})-0.84{ln}^{2}(\omega_{1}/\omega_{2})$$

(6) $$\omega =\frac{A_{p}\sigma_{p0}+A_{r}f_{y}}{f_{ck}bd_{p}}$$

and where *ω* is the combined reinforcing index; the subscripts 1 and 2 represent the midspan and center support, respectively; *d_p_* is the tendon depth; and *f_y_* is the yield strength of steel rebars.

**Figure 15:**
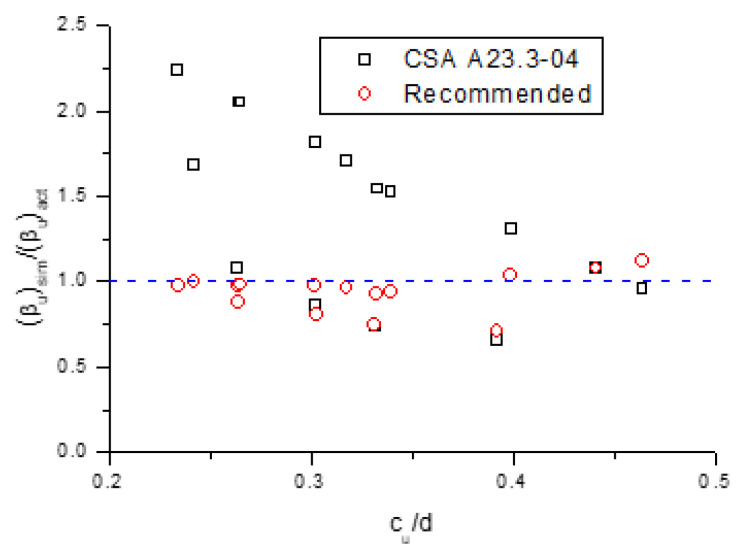
Values of (*β_u_*)_sim_/(*β_u_*)_act_ against *c_u_*/*d* according to CSA A23.3-04 and recommended equations.

Figure 15 shows the variation in (*β_u_*)_sim_/(*β_u_*)_act_ against *c_u_*/*d* for the 15 investigated beams with different types of rebars, where (*β_u_*)_sim_ represents the moment redistribution calculated from the simplified equations (i.e., CSA A23.3-04 and recommended), and (*β_u_*)_act_ represents the actual moment redistribution generated by the numerical analysis. We see that the recommended equation is substantially more accurate than CSA A23.3-04 for quantifying moment redistribution in these beams. In addition, the data by the recommended equation are mostly on the safe side (i.e., (*β_u_*)_sim_/(*β_u_*)_act_ < 1). In contrast, most of the predictions by CSA A23.3-04 are unsafe (i.e., (*β_u_*)_sim_/(*β_u_*)_act_ > 1).

## 5. Conclusions

An investigation was carried out to evaluate the effect of adopting FRP instead of steel rebars on moment redistribution in continuous PCBs with external CFRP tendons. The main conclusions of the investigation are as follows:

- Moment redistribution in the beams with FRP rebars was contributed by concrete cracking, and tended to stabilize after the stabilization of crack evolution. For the beams with steel rebars, apart from the contribution by concrete cracking, steel yielding led to further development of moment redistribution;
- Steel rebars led to significantly higher redistribution of moments than FRP rebars. The redistribution difference between the beams with steel and FRP rebars enlarged with increasing *ρ_r_*_2_ up to 1.67% and decreased thereafter;
- The current codes of practice investigated (Eurocode 2, CSA A23.3-04 and ACI 318-19) could not reflect the influence of both the amount, and type, of rebars on moment redistribution in PCBs with external tendons. In addition, it was found that the codes may lead to unsafe predictions in moment redistribution in beams with FRP rebars;
- A simplified equation was recommended to predict moment redistribution in externally PCBs with steel and FRP rebars. It was shown that the recommended equation yields accurate and conservative predictions.

## Data Availability

Not applicable.
